# A father collaborates with a research professor to tell the father's story. What could we ALL have done better? Donald looks back at his daughter's eating disorder

**DOI:** 10.1186/2050-2974-2-S1-O52

**Published:** 2014-11-24

**Authors:** Donald Irvine, Michael P Levine

**Affiliations:** 1 F.E.A.S.T, Perth, Australia; 2 Department of Psychology Kenyon College, Gambier, United States

## 

A Father/Dad/Husband story of a 13 year Eating Disorder (AN) experience, pre-diagnosis, post diagnosis/pre/post major relapse, into recovery.

A father's story of what is it like to experience a 13 year old caring, loving, bubbly, confident, talented, high/achieving & intelligent girl starting her teenage years transform into a 16 year old, struggling with feelings & thoughts, becoming a teenager centred on food intake & body image with personality changes. As a parent, what does it feel like seeing her talents, intelligence & bubbliness fade, becoming a 16 year old, physically fading & having increasing difficulty with school & studies, & family/friends?

In this presentation Donald Irvine with the collaboration of Professor Michael Levine will describe his family's journey & experiences, including his own, as; (1) his daughter became ill with an eating disorder; (2) as the disorder progressed and enveloped the family, including a significant relapse; and (3) as the family struggled to locate and participate in treatment & finding recovery.

Donald' will conclude by sharing some of the hard-earned lessons & successes from this ongoing journey, in the hope that his experiences will be of benefit to sufferers, carers, and treatment providers.

This abstract was presented in the **Learning from Consumers** stream of the 2014 ANZAED Conference.

